# A perspective on the role of class III semaphorin signaling in central nervous system trauma

**DOI:** 10.3389/fncel.2014.00328

**Published:** 2014-10-27

**Authors:** Vasil Mecollari, Bart Nieuwenhuis, Joost Verhaagen

**Affiliations:** ^1^Laboratory for Regeneration of Sensorimotor Systems, Netherlands Institute for NeuroscienceAmsterdam, Netherlands; ^2^Department of Molecular and Cellular Neurobiology, Center for Neurogenomics and Cognitive Research, Neuroscience Campus Amsterdam, VU University AmsterdamAmsterdam, Netherlands

**Keywords:** class III semaphorins, neuropilins, plexins, central nervous system trauma, axonal regeneration, re-vascularization, immune response, re-myelination

## Abstract

Traumatic injury of the central nervous system (CNS) has severe impact on the patients’ quality of life and initiates many molecular and cellular changes at the site of insult. Traumatic CNS injury results in direct damage of the axons of CNS neurons, loss of myelin sheaths, destruction of the surrounding vascular architecture and initiation of an immune response. Class III semaphorins (SEMA3s) are present in the neural scar and influence a wide range of molecules and cell types in and surrounding the injured tissue. SEMA3s and their receptors, neuropilins (NRPs) and plexins (PLXNs) were initially studied because of their involvement in repulsive axon guidance. To date, SEMA3 signaling is recognized to be of crucial importance for re-vascularization, the immune response and remyelination. The purpose of this review is to summarize and discuss how SEMA3s modulate these processes that are all crucial components of the tissue response to injury. Most of the functions for SEMA3s are achieved through their binding partners NRPs, which are also co-receptors for a variety of other molecules implicated in the above processes. The most notable ligands are members of the vascular endothelial growth factor (VEGF) family and the transforming growth factor family. Therefore, a second aim is to highlight the overlapping or competing signaling pathways that are mediated through NRPs in the same processes. In conclusion, we show that the role of SEMA3s goes beyond inhibiting axonal regeneration, since they are also critical modulators of re-vascularization, the immune response and re-myelination.

## Highlights

- Class III semaphorins (SEMA3s), apart from their classical axon repulsive properties, exert regulatory functions in a variety of non-neuronal cells associated with CNS trauma.
- Neuropilins (NRPs) are pleiotropic receptors involved in multiple signaling pathways controlling tissue remodeling following CNS trauma.
- Semaphorin signaling is regulated at several levels, including receptor complex formation, proteolytic cleavage and interaction with extracellular matrix molecules.
- Interfering with class III semaphorin signaling might be beneficial for axonal re-growth, re-vascularization, re-myelination and manipulation of the immune response following CNS trauma.

## Introduction

The semaphorin family consists of secreted and transmembrane glycoproteins that are involved in many cellular functions. Semaphorins (SEMAs) are subdivided into eight classes based on amino acid sequence similarities and structural features. The first two classes of SEMAs are found in invertebrates, class III till VII belong to vertebrates, while class VIII semaphorins are only expressed by viruses (Semaphorin Nomenclature Committee S. N., 1999). The first semaphorin, at that time named fasciclin IV, was identified in the grasshopper and was later renamed semaphorin1a (Kolodkin et al., 1992). SEMA3s are the best described SEMAs and, like semaphorin1a, were initially identified by neurobiologists in an effort to identify proteins with an effect on axon fasciculation, guidance and growth cone steering (reviewed in Tran et al., 2007; Yoshida, 2012) and, somewhat later as factors that affect neuronal polarization and synapse formation during the development of the nervous system (reviewed in Pasterkamp, 2012; Tillo et al., 2012).

The first member of SEMA3s, at that time designated collapsin-1 and now referred to as Sema3A, was isolated from the chick brain (Luo et al., 1993) and it was shown to induce collapse of dorsal root ganglion (DRG) growth cones. This characteristic gave rise to one of the most widely used bioassays to study chemorepulsive proteins, the DRG growth cone collapse assay. There are currently seven members of secreted SEMA3s, named SEMA3A through SEMA3G. Their signaling is mediated via heterodimer receptor complexes that contain neuropilins as specific binding sub-units and plexins (PLXNs) as signal transduction sub-units. The compositions of receptor complexes differ per SEMA3 member (reviewed in Sharma et al., 2012). However, when functional receptor complexes are formed, downstream signaling cascades are activated that eventually propagate SEMA3 biological responses.

Apart from the assorted binding specificity for NRPs and PLXNs, SEMA3 signaling is modulated in a very complex and diverse fashion. Firstly, the binding affinity of SEMA3s for NRPs is regulated by furin dependent proteolytic cleavage resulting in forms of SEMAs with different activity (Adams et al., 1997; Parker et al., 2010, 2013; Guo et al., 2013). Secondly, SEMA3s signaling is modulated by competition with other molecules that signal via NRPs. For instance, NRPs act as co-receptors for the vascular endothelial growth factor (VEGF) family (Soker et al., 1998; Gluzman-Poltorak et al., 2000; Whitaker et al., 2001; Favier et al., 2006; Prahst et al., 2008; Herzog et al., 2011), transforming growth factors-β (TGF-β; Glinka and Prud’homme, 2008; Glinka et al., 2011) and other growth factors such as epidermal growth factor (EGF; Rizzolio et al., 2012) and platelet derived growth factor (PDGF; Banerjee et al., 2006; Ball et al., 2010; Cao et al., 2010; Pellet-Many et al., 2011). Moreover there are indications that NRPs are able to interact directly with members of the integrin family of surface receptors (Fukasawa et al., 2007; Valdembri et al., 2009) in addition to the previously reported regulation of integrin functions indirectly by SEMA3 signaling (Barberis et al., 2004; Kruger et al., 2005; Serini et al., 2008). Adding even more complexity to this wide array of interactions, various molecules control the NRP-PLXNA receptor complex. For example, the interaction of the complex with L1 cell adhesion molecules (L1CAMs) or neuronal cell adhesion molecules (NrCAMs) can modulate SEMA3 signaling towards repulsion or attraction during axon guidance (Castellani et al., 2000, 2002; Falk et al., 2005). Collectively the observations above demonstrate that the signaling activity of SEMA3s can be regulated at multiple levels, including proteolytic processing of SEMA3s, interaction with other ligands and variation in receptor composition.

SEMA3s signaling plays a significant role following traumatic central nervous system (CNS) injuries due to their presence in neural scar tissue. The expression of SEMA3s following CNS trauma is suggested to be important in both the acute and sub-acute/chronic phases of scar formation. To date there is data showing Sema3A mRNA up-regulation as early as 1 day after CNS penetrating lesions (Pasterkamp et al., 1999), which becomes more prominent at 7 days post-axotomy and persists for up to 2 months. In the above study, a significant increase in Sema3A mRNA expression co-localized with several fibrotic markers but not with glial cell or blood derived cell markers. De Winter et al. (2002) validated these findings and expanded them with mRNA expression patterns for more SEMA3 members, namely Sema3B, C, E and F. The expression profile and time-course for the other SEMA3s was similar to the findings from Pasterkamp et al. (1999), however Sema3B expression was additionally detected in S100 positive cells in and around the lesion area, while a marked difference in expression patterns was observed between the transected and contused spinal cord.

Other studies have reported a somewhat different Sema3A mRNA expression pattern following spinal cord transection. Hashimoto et al. (2004) found that Sema3A transcripts were swiftly reduced in NeuN positive cells 24 h post spinal cord transection and reached 80% of normal expression levels at 28d. However the same study failed to detect co-localization of Sema3A with fibronectin (FN), in contradiction with most other studies. The detection of SEMA3s at the protein level has been hampered for a long time from the lack of reliable SEMA3 antibodies. Nevertheless, with new commercial antibodies becoming available, the expression of Sema3A protein has since been demonstrated both in brain (Minor et al., 2011) and spinal cord injuries (SCI; Mire et al., 2008). Sema3A was consistently found in the fibrotic component of the scar indicating that the main source are the meningeal cells invading the lesion core, in line with previous findings on the localization of the SEMA3 mRNAs (Pasterkamp et al., 1999; Niclou et al., 2003). There are indications that SEMA3A and SEMA3F might also be expressed from glial cells under stress conditions as shown in chronic phases of multiple sclerosis (MS) lesions (Williams et al., 2007; Piaton et al., 2011; Boyd et al., 2013).

On the other hand, the presence and source of SEMA3s during the acute phase following induction of CNS trauma has been until recently unsubstantiated. In earlier studies Sema3A mRNA was localized in the vicinity of blood vessels, implying that disruption of the vasculature causes a temporal upregulation of this protein (Pasterkamp et al., 1999). However, the lack of co-localization with several blood-derived cell markers indicates that the inclusion of additional cell markers is of critical importance for future studies. More recently, there is a distinct line of evidence which indicates that SEMA3s are expressed *in vitro* in human peripheral blood monocytes and monocyte-derived M2-like macrophages (Ji et al., 2009) and from T-cells and dendritic cells (DCs) upon activation by inflammatory cytokines (Lepelletier et al., 2006). These findings corroborate *in vivo* observations in brain injuries, where Sema3A was found to be expressed in the extracellular space after focal cerebral ischemia after 2 h, 4 h and 8 h of reperfusion (Jiang et al., 2010). Additionally in a similar experimental stroke model, Sema3A was upregulated 1 day following injury onwards and partially co-localized with endothelial and neuronal cells (Pekcec et al., 2013). Finally, a potential source of SEMA3s in the acute phase might be the neurons themselves, since ischemic neurons are known to secrete Sema3A in response to hypoxia conditions affecting both microglia functions (Majed et al., 2006) and revascularization efficacy (Joyal et al., 2011). Consequently, it is currently well established that SEMA3s are highly expressed in the acute and subacute/chronic phases of CNS trauma. As discussed below this may have obvious implications for neural scar tissue remodeling.

The neural scar is a complex tissue that consists of many cell types including, astrocytes and other glial cells, various blood-borne cells, fibroblast, and neural precursor cells, and thus it constitutes a physical and molecular barrier that can block nerve regeneration (reviewed in Silver and Miller, 2004). SEMA3s are regarded as one of the major classes of axon repulsive molecules that contribute to the failure of axons to regenerate through the neural scar. Apart from their direct influence on axonal regeneration (reviewed in Pasterkamp and Verhaagen, 2006), there is a wealth of data in the literature that suggests a role for SEMA3s and their receptors in the modulation of the immune response (reviewed in Mizui et al., 2009; Takamatsu and Kumanogoh, 2012; Kumanogoh and Kikutani, 2013), re-vascularization (reviewed in Geretti et al., 2008; Neufeld and Kessler, 2008; Sakurai et al., 2012) and re-myelination (reviewed in Kotter et al., 2011). The aim of this literature review is to highlight these additional functions of semaphorin signaling and to discuss these in the context of the injured adult CNS.

## The role of semaphorin signaling in axonal regeneration

Traumatic CNS injury has a severe impact on all cell types in the injured neural tissue. One of the major obstacles for regeneration is that axons of CNS neurons do not re-grow after injury. The poor intrinsic neuronal growth capacity of CNS neurons and the inhibitory extrinsic environment contribute to the failure of axonal regeneration (reviewed in Afshari et al., 2009). Three major classes of axon repulsive molecules are identified at the site of injury: (1) myelin-derived axon repulsive molecules; (2) chondroitin sulfate proteoglycans (CSPGs); and (3) classical repulsive axon guidance molecules. The best-characterized myelin-derived axon repulsive molecules are reticulon 4 (Nogo-A), myelin associated glycoprotein (MAG) and oligodendrocyte-myelin glycoprotein (OMgp) (reviewed in Xie and Zheng, 2008). CSPGs located in the extracellular matrix of the glial scar are structurally modified and drastically up regulated after traumatic CNS injury. Most isoforms of CSPGs restrict axonal regeneration (reviewed in Kwok et al., 2011). Over and above, classical axon guidance molecules including ephrins, slits, wnts, and SEMAs limit neural regeneration following injury (reviewed in Harel and Strittmatter, 2006; Niclou et al., 2006; Pasterkamp and Verhaagen, 2006; Giger et al., 2010). From the latter, SEMA3s have been extensively studied due to their predominant inhibitory properties for axonal outgrowth during the development of the CNS.

Several studies interfered with distinct inhibitory components of the neural scar after CNS injury with the rationale to overcome the axon repulsive environment and stimulate axonal regeneration and functional recovery. Axon repulsive molecules in the extracellular matrix are commonly targeted by the bacterial enzyme chondroitinase ABC (ChABC) and a small CSPG called Decorin. ChABC digests the chondroitin sulphate (CS)— glycosaminoglycan (GAG) chains of CSPGs, while decorin suppresses multiple repulsive proteoglycans in the extracellular matrix. Among the repulsive molecules that could be affected from treatment with the above approaches is SEMA3A which in earlier *in vitro* studies was shown to be removed from the surface of cultured neuronal cells upon ChABC treatment (De Wit et al., 2005). Later *in vivo* evidence demonstrated that the association of Sema3A to the ECM can indeed be reduced by ChABC (Dick et al., 2013; Vo et al., 2013) and decorin treatments (Minor et al., 2011). This is particularly interesting since both approaches have been reported to be beneficial for functional recovery after CNS injury (Bradbury et al., 2002; Davies et al., 2004; Soleman et al., 2012 and reviewed in Kwok et al., 2011). The ChABC and Decorin studies confirmed that targeting the CSPGs and associated repulsive components in the extracellular matrix results in a more permissive environment for injured axons to re-grow. Thus, interference with repulsive axon guidance molecules in the scar such as SEMA3s may be a potential treatment for traumatic CNS injuries.

Accordingly, several studies aimed to block neuronal sensitivity to SEMA3s by modulating multiple semaphorin receptor complexes. Application of an L1 mimetic peptide prevented the effects of Sema3A on axon growth inhibition and growth cone collapse *in vitro*, but was unable to enhance axonal regeneration and functional recovery after SCI *in vivo* (Mire et al., 2008). Knock-out of multiple receptor components for Nogo-A, MAG and Sema3s in mice was unable to enhance regeneration of the axons of serotonergic neurons after complete spinal cord transection. The axons of mice that are deficient in Nogo receptor 1 (NgR1) and the semaphorin signal transducing receptors PLXNA3 and PLXNA4 were unable to penetrate through the repulsive environment of the neural scar (Lee et al., 2010). This would suggest that other extrinsic factors in the scar tissue, in addition to myelin-derived axon repulsive molecules and SEMA3s are capable to restrict axonal regeneration.

On the other hand inhibition of SEMA3A itself seems like a more efficient approach. A peptoid called semaphorin induced chemorepulsion inhibitor (SICHI) enhances axonal regeneration in an *in vitro* axotomy model of adult hippocampal slices (Montolio et al., 2009). *In vivo*, anti-Sema3 antibody treatment rescues retinal ganglion cells (RGCs) from Sema3A-induced apoptosis after optic nerve axotomy (Shirvan et al., 2002). Another Sema3A inhibitor called Xanthofulvin (SM-216289) (Kumagai et al., 2003) strongly interferes with NRP1 activation and consequently the Sema3A induced growth-cone collapse and repulsive activity in embryonic DRG explants. Furthermore, chronic administration of Xanthofulvin enhanced olfactory nerve regeneration after axotomy *in vivo* (Kikuchi et al., 2003) and promoted a regenerative response and functional recovery after a complete spinal cord transection (Kaneko et al., 2006). More specifically, the inhibition of Sema3A reduced apoptosis, decreased the cavity volume of the scar, increased the number of regenerating fibers expressing NRP1 into the scar, led to robust Schwann cell migration into the lesion, promoted re-myelination, and enhanced angiogenesis. However it should be noted that Xanthofulvin is a potent Sema3A inhibitor, but additionally interferes with the signaling pathways of ephrins, epidermal growth factor receptors (EGFRs) and fibroblast growth factor receptors (FGFRs; Kaneko et al., 2006 suppl.). Collectively, the latter studies demonstrated that Sema3A contributes to the failure of CNS axonal regeneration *in vivo* and additionally interferes with cellular responses that could support recovery after CNS trauma.

SEMA3s have a high affinity for NRPs that act as co-receptor for multiple ligands. One of the other molecules that signals via NRPs are VEGFs (reviewed in Olsson et al., 2006) and compete with SEMA3s for NRP binding (Gu et al., 2002; Appleton et al., 2007). VEGFs are crucial players in angiogenesis (as discussed in the next section on re-vascularization) and have been suggested to also exert a direct neuroprotective role. The first evidence for a neuroprotective role of VEGFs came from an *in vitro* model of cerebral ischemia. VEGF rescued cultured CNS neurons from hypoxia and glucose deprivation induced apoptosis (Jin et al., 2000). Accordingly, *in vivo* administration of VEGF in the acute phase after focal ischemic brain injury, prevented neuronal cell death and was found to enhance neurogenesis in chronic stages (Sun et al., 2003). This is of particular interest since in a similar *in vivo* situation Sema3A is implicated in cerebral ischemia-induced neuronal death (Jiang et al., 2010) highlighting the dual ligand interaction capacity of NRP1-signaling following brain injury.

Taken together the above experimental data indicate that SEMA3s are present in scar tissue and have a negative impact on axonal regeneration by inducing apoptosis and repulsion of axons of CNS neurons. Several studies have demonstrated that targeting the SEMA3s receptors at injured neurons alone is insufficient to promote axonal regeneration. However, inhibition of Sema3A itself was shown to improve various regenerative processes. In addition to their direct effects, SEMA3s compete with multiple ligands for NRP binding including the neuroprotective VEGFs. Therefore it cannot be ruled out that by directly targeting SEMA3s, the binding sites at NRPs become available for interaction with VEGFs for example thus offering yet another mechanism that may prevent neural apoptosis. Furthermore, direct targeting of SEMA3s could influence the complex process of tissue remodeling in and around the lesion area as outlined further in this review. Disruption of the vascular architecture, the inflammatory response and the loss of axonal myelin sheaths following CNS trauma are factors that additionally influence neural tissue and functional recovery and appear to be sensitive to SEMA3 signaling. From this perspective, the impact of blocking semaphorin signaling following CNS trauma goes beyond influencing axonal guidance and regeneration.

## The role of semaphorin/VEGF signaling in re-vascularization

One of the hallmarks of CNS trauma is damage to the surrounding vascular architecture. Acute rupture of the blood vessels after CNS injury initiates a secondary injury response that drastically limits the regenerative capacities of injured CNS neurons (reviewed in Oudega, 2012). Firstly, the decreased blood supply causes ischemia-induced apoptosis of neurons and glial cells (Casella et al., 2006). Secondly, breakdown of the blood-brain barrier (BBB)/blood-spinal cord barrier (BSCB) results in oedema formation. Furthermore, the increase in vascular permeability allows foreign molecules and inflammatory cells to enter the injury site (reviewed in Engelhardt and Coisne, 2011). The permeability of the BSCB is maximal around 24 h following SCI but is gradually restored to normal after 14 days (Figley et al., 2014). The BBB permeability after traumatic brain injury may differ from BSCB permeability after spinal injury due to morphological differences (reviewed in Bartanusz et al., 2011). However, the re-establishment of new blood vessels following CNS injury, often referred to as angiogenesis or re-vascularization, is of key importance for functional recovery after a CNS injury.

The ischemic and hypoxic conditions after traumatic CNS injury triggers re-vascularization by up-regulating angiogenic growth factors. However, new blood vessels are mainly formed during the first week of injury (Figley et al., 2014) indicating that the endogenous angiogenic capacity is mostly restricted to the early phase following CNS injury. The VEGF family constitutes the key angiogenic growth factors since they stimulate blood vessel growth by endothelial cell (EC) migration, survival and proliferation. The mammalian VEGF family consists of five secreted glycoproteins named VEGFA, B, C, D and placental growth factor (PGF). Alternative splicing of the mRNAs encoding the VEGF members results in a plethora of splice variants with different activity and binding affinities for receptors (reviewed in Olsson et al., 2006; Adams and Eichmann, 2010). The splice variants of VEGFA are secreted by various cell types at the injury site including astrocytes (Bartholdi et al., 1997; Herrera et al., 2009), microglia (Chen et al., 2013) and invading inflammatory cells (Sköld et al., 2000).

VEGFA is extensively investigated in re-vascularization due to the rapid upregulation of this VEGF member and its receptors after CNS injury (Sköld et al., 2000; Pugh and Ratcliffe, 2003; Widenfalk et al., 2003; Chen et al., 2013). Notably, the observed drop in VEGFA expression during later stages of recovery after CNS trauma (Sköld et al., 2000; Vaquero and Zurita, 2004; Herrera et al., 2009) correlates with the limited endogenous angiogenesis capacities. This suggests that VEGFA is important for the endogenous re-vascularization early after CNS injury and thus became the focus of research in therapeutic angiogenesis, which aims to extend the time window of blood vessel formation in order to improve blood supply and neuronal survival after CNS trauma. Application of VEGFA and more specifically its VEGF_165_ splice variant was shown to stimulate angiogenesis after CNS trauma in brain (Sun et al., 2003; Zechariah et al., 2013) and spinal cord (Facchiano et al., 2002; Widenfalk et al., 2003; De Laporte et al., 2011). However, it is still controversial whether VEGFA/VEGF_165_ treatment improves or aggravates neural and functional recovery (reviewed in Kundi et al., 2013). The results of recent studies are summarized in Table 1. This controversy stems majorly from differences in study design that could greatly influence the experimental outcome, while on the other hand it should be noted that re-vascularization after injury is regulated by multiple EC growth factors, including the other members of the VEGF-family, e.g., angiopoietins, fibroblast growth factors (FGFs) and PDGFs (Benton et al., 2009; Carmeliet and Jain, 2011; Lieu et al., 2011). Hence, other modulators of angiogenesis may interfere with the angiogenic potential of VEGFA treatments particular in chronic stages of traumatic CNS injury. We hypothesize that one mechanism could be that injury induced SEMA3s compete with VEGFs during the (sub)chronic phases of CNS trauma.

**Table 1:** **Summary of experimental studies that assessed the therapeutic angiogenic potential of VEGF after CNS trauma**.

Growth factor supplied	Injury model	Time of delivery	Delivery method	Main findings	Reference
VEGF_165_	spinal cord hemisection		gelfoam with growth factor	- improves angiogenesis - enhances tissue sparing - promotes axonal sprouting	Facchiano et al. (2002)
	spinal cord contusion	acute (immediately following injury)	single injection	- improves angiogenesis - enhances tissue sparing - reduces apoptosis - improves locomotor recovery up to 6 weeks after injury	Widenfalk et al. (2003)
				- reduces cavity formation - enhances tissue sparing	Sundberg et al. (2011)
				- no effect on locomotor recovery - increases incidence of allodynia	
				- no effect on tissue sparing	Herrera et al. (2009)
			gelfoam with growth factor	- elevates BSCB permeability - improves locomotor recovery up to 4 weeks but diminishes on 8 weeks after injury	Patel et al. (2009)
		acute - subchronic (0–7 days post injury)	daily injections	- reduces mechanosensitivity	van Neerven et al. (2010)
				- no effect on thermal sensitivity - no effect on motor functions - no effect on tissue sparing	
		subchronic (3 days post injury)	single injection	- no effect on angiogenesis - increases vascular permeability - increases leukocytes infiltration - exacerbates histopathology	Benton and Whittemore (2003)
VEGF	focal cerebral ischemia	subchronic - chronic (3–21 days post injury)	daily delivery by osmotic pumps	- improves angiogenesis - reduces infarct volume - reduces inflammatory response - enhances pericyte functions	Zechariah et al. (2013)
		subacute - subchronic (1–3 days post injury)		- improves angiogenesis - reduces infarct volume - acute neuroprotective effects - improves neurological performances	Sun et al. (2003)
VEGF_165_ and FGF2	spinal cord hemisection	acute -subchronic (0–7 days post injury)	implantation of protein loaded channel bridges	- improves angiogenesis	De Laporte et al. (2011)
				- no effect on axonal regrowth	

*This table highlights the opposing effects of VEGFA administration after CNS trauma reported by different laboratories. The contradiction in the outcome of VEGF treatment may arise from technical differences in study design, while the different assessment approaches do not allow for direct comparison of the studies. In this table, the literature on VEGF is organized according to type of VEGF applied, the injury model, time of delivery and the delivery method. The main findings of each paper are summarized and the colors indicate whether the results are considered to be positive (green), absent or negative (red). All studies used rat as experimental animal except Zechariah et al. (2013) who used mice*.

VEGF signaling is mediated by tyrosine kinase receptors (VEGFR1, VEGFR2, and VEGFR3) and receptor complexes consisting of NRPs and VEGFRs (Gluzman-Poltorak et al., 2000; Whitaker et al., 2001; Favier et al., 2006). Activation of NRP-VEGFR complexes lead to enhanced VEGF signaling in ECs (Soker et al., 1998; Herzog et al., 2011). Blockage of endogenous VEGF_165_ binding to NRP1 reduces vascular permeability and micro-hemorrhage formation after 24 h of experimentally induced BBB disruption in mice (Suidan et al., 2012). Knock-in mice expressing a mutated NRP unable to bind VEGF_165_, are viable but develop post-natal angiogenesis defects in hearth and retina (Fantin et al., 2014). This indicates that NRP1 is an essential component of VEGF signaling and that growth factors other than VEGF_165_ might also contribute to angiogenesis and re-vascularization. Similar to VEGFs, SEMA3s are up-regulated after traumatic CNS injury and their signaling is mediated via NRP complexes. Recent findings suggest that SEMA3s play an inhibitory role in re-vascularization after injury thus suggesting a potential interplay between SEMA3s and VEGF after neural trauma.

Classical axon guidance molecules, including SEMA3s and their receptors, play an import role in the development of the vascular system (Carmeliet and Tessier-Lavigne, 2005). Injury induced SEMA3s may act directly on ECs and inhibit revascularization. One of the working hypotheses is that SEMA3s compete with VEGF_165_ for binding to NRPs at ECs. The functional competition between both ligands arises by overlap in the binding areas at the NRP receptors. The NRP b1b2 domains are essential for VEGF_165_ binding (Herzog et al., 2011), while SEMA3s requires both the a1a2 and b1 domains for NRP interaction (Gu et al., 2002; Geretti et al., 2007, 2008; Herzog et al., 2011). Moreover, the stereological conformation of the NRPs allows only one type of ligand interaction. Indeed, Sema3s were found to compete with VEGFs for neuropilin receptors in binding assays *in vitro*. Sema3A is a competitor for VEGF_165_ at NRP1 (Miao et al., 1999); while Sema3F interferes with VEGF_165_ binding at both NRP1 and NRP2 (Geretti et al., 2007; Parker et al., 2010). In line with these structural observations, Sema3A and Sema3F inhibit VEGF_165_ induced ERK1/2 activity and EC proliferation *in vitro* (Kessler et al., 2004; Guttmann-Raviv et al., 2007). Moreover, Sema3A interferes with the chemotactic properties of VEGF_165_ on ECs expressing NRP1 *in vitro* (Miao et al., 1999) and VEGF_165_-induced angiogenesis *in vivo* (Acevedo et al., 2008). Taken together, the characteristic structure of NRPs and the functional competition between SEMA3s and VEGF_165_ support a role for SEMA3s as inhibitors of the pro-angiogenic effects of VEGF_165_.

Alongside the direct competition with VEGFs for NRP binding, semaphorin signaling itself has a wide spectrum of anti-angiogenic effects. The role of SEMA3s and their receptors in angiogenesis have been studied in cell cultures, cancer tissue and in the CNS related to pathology of the retina. Various assays of cultured ECs demonstrated that SEMA3s affect the function of ECs. Sema3A inhibits cell motility and initiates the collapse of the cytoskeleton of ECs expressing NRP1 (Miao et al., 1999). Similarly, human recombinant SEMA3F induces cytoskeleton collapse of EC expressing NRP2 (Shimizu et al., 2008). Therefore, Sema3A and SEMA3F are chemorepulsive for ECs and inhibit their proliferation in culture. Interestingly, these effects do not depend on competition for NRPs binding with VEGF_165_ as shown in cultures that do not contain this growth factor. Most importantly, the repulsive and anti-proliferation effects of Sema3A and SEMA3F on EC are synergistic *in vitro*. The expression levels of different SEMA3s after injury may therefore be important since high concentrations of Sema3A and SEMA3F induces ECs apoptosis *in vitro* (Kessler et al., 2004; Guttmann-Raviv et al., 2007).

In addition to Sema3A and SEMA3F, SEMA3B affect ECs that express NRP1 and NRP2. SEMA3B signaling repels ECs, inhibits cell adhesion, causes collapse of the cytoskeleton, reduces VEGF_165_ induced ERK1/2 phosphorylation and apoptosis *in vitro* (Varshavsky et al., 2008). Notably, Varshavsky et al. demonstrated that furin dependent proteolytic cleavage of SEMA3B reduces the activity of this semaphorin and its effects on ECs. Moreover, transcriptome screening of the ECs *in vitro* and in mammalian tissue demonstrated that another SEMA3, Sema3G is expressed in vascular tissue (Kutschera et al., 2011). The latter study furthermore showed that recombinant Sema3G stimulates denudation (clearance of surface receptors) of ECs that were cultured on smooth muscle cells. This suggests that Sema3G stimulates EC functions to form new blood vessels. Collectively, SEMA3s affect the function of ECs directly (independent of VEGF) and may have an inhibitory role in angiogenesis. However, one exception appears to be Sema3G that serves as a positive regulator of angiogenesis *in vitro*.

Even though there is an explicit need in investigating SEMA3s in the injured nervous system in the context of re-vascularization, to date the majority of evidence originates from the field of cancer biology. In this field of research, it is evident that several SEMA3s concomitantly target endothelial and cancer cells and can strongly restrict angiogenesis and tumor growth, rendering them a principal anti-tumor therapeutic target (reviewed in Tamagnone, 2012). Accordingly, the seven members of SEMA3s and NRP receptors are heterogeneously expressed at the mRNA level in many human glioma tumors (Karayan-Tapon et al., 2008). Several studies investigated the effects of SEMA3s on tumor angiogenesis *in vivo*. Tumors that express SEMA3A (Maione et al., 2009; Casazza et al., 2011) SEMA3B (Rolny et al., 2008), SEMA3D (Sabag et al., 2012), Sema3E (Sakurai et al., 2010) or SEMA3F (Bielenberg et al., 2004; Kessler et al., 2004) have a disorganized vesicular architecture compared to controls. Most of the tumors that express SEMAs have fewer, smaller or less branched blood vessels. Blood vessels in tumors expressing SEMA3B have deficits in the recruitment of pericytes, which normally stabilize EC tube formation (Rolny et al., 2008). In contrast to the *in vivo* findings reported from Kutschera et al., SEMA3G inhibits angiogenesis in experimentally generated tumors *in vivo*, which were derived from a glioblastoma cell line and implanted in the cortex (Sabag et al., 2012). Thus, SEMA3 signaling in tumors influences ECs and pericytes and is inhibitory for tumor angiogenesis *in vivo*.

Semaphorins also affect re-vascularization in the CNS. In proliferative retinopathies (PRs), there is degeneration of the vascular architecture and the hypoxic regions display dysregulated hyper revascularization. Interestingly, the newly formed blood vessels are not capable to reach the neural retina. The inflammatory and ischemic stress induced expression of Sema3A by RGCs prevented revascularization of the eye since RGC-derived Sema3A repelled blood vessels and inhibited EC growth (Joyal et al., 2011). In addition, Sema3A signaling via NRP1 causes loosening of EC junctions leading to vascular hyper permeability and macular edema *in vivo* (Cerani et al., 2013). Sema3A is unique among SEMA3s since it induces vascular permeability via NRP1 (Acevedo et al., 2008). On the other hand, inhibition of CNS angiogenesis appears to be mediated also by Sema3E that signals via PlexinD1, in a NRP1-independent fashion. In particular, ischemic neurons secrete Sema3E, which mediates the retraction of endothelial filopodia by activating small GTPases like RhoJ. In a mouse model of ischemic retinopathy, silencing of PLXND1 signaling enhanced re-vascularization at the extraretinal area of the eye. Consistently, the number of extraretinal vessels was decreased following intravitreal injection of Sema3E protein (Fukushima et al., 2011). This demonstrates that Sema3E and PLXND1 signaling inhibits angiogenesis in ischemic retinopathy *in vivo*.

At least part of the observed SEMA3s functions are mediated through regulation of integrins. Integrin receptors are located on the cell surface of ECs and have a major role in angiogenesis by promoting EC adhesion, migration and survival. There are twenty-four unique heterodimers of integrin receptors that bind to a wide range of ligands in the extracellular matrix (reviewed in Avraamides et al., 2008). SEMA3 signaling can modulate integrin receptors at ECs and thereby influence angiogenesis. Sema3A and Sema3F inhibit integrin-mediated adhesion and migration of cultured ECs on FN and vitronectin (VN) substrates. Accordingly, interference with NRP1 and PLXNA1 signaling enhanced adhesion and migration on these integrin ligands (Serini et al., 2003). In addition to Sema3A and Sema3F, Sema3E suppresses the function of integrins and could lead to loss of integrin-mediated adhesion to the extracellular matrix (Sakurai et al., 2010). The same study revealed some of the mechanisms how Sema3E negatively regulates the availability of integrin receptors at the cell surface. Firstly, Sema3E signaling via PLXND1 leads to inactivation of Ras**-**related protein (R-Ras), which in turn, inactivates integrin receptors. Secondly, Sema3E induces the activation of ADP-ribosylation factor 6 (Arf6) positive vesicles that promote internalization of integrin receptors. In contrast to these functions of SEMA3s as inhibitors of angiogenesis and integrin functions, Sema3C promotes angiogenesis *in vitro* by activation of integrins (Banu et al., 2006). Collectively, the aforementioned studies propose yet another mechanism on how SEMA3s might affect re-vascularization, namely by modulating the function of integrins.

Reestablishing the metabolic supply to neurons may accelerate regeneration and could be required for functional recovery after traumatic CNS injury. The opposing functions of VEGFA and SEMA3s on ECs may limit the endogenous angiogenic capacities after CNS trauma. The relative expression of VEGFA and SEMA3s over time could therefore drastically influence re-vascularization, where VEGFA would be beneficial and Sema3s would limit vascularization. VEGFA is highly expressed in early re-vascularization stages and contributes to endogenous repair of the vascular architecture. In the sub-chronic and chronic stages, VEGFA expression declines while the expression of SEMA3s rises. The injury-induced expression of SEMAs could compromise vascular repair after CNS trauma. Firstly, SEMA3s interfere with the VEGFA pro-angiogenesis effects by direct competition for NRPs. SEMA3s could additionally modulate bFGF and PDGF signaling, via NRPs, that also influence angiogenesis. Secondly, SEMA3s expressed by ischemic neurons and meningeal fibroblasts could have a wide spectrum of anti-angiogenesis effects by regulating EC adhesion, migration, proliferation, survival and recruitment of pericytes. The effects of SEMA3s on angiogenesis may be mediated or depend on the modulation of cell surface receptors such as integrins. Future studies should focus on the question whether inhibition of various SEMA3s could therefore promote revascularization and additionally make the neural scar more permissive for regenerating axons. Kaneko et al. (2006) showed that application of the sema3A inhibitor Xanthofulvin in the injured spinal cord indeed promoted axonal regeneration and angiogenesis at the lesion site. This observation is in line with the inhibitory role of SEMA3s on re-vascularization after traumatic CNS injury.

## The role of semaphorin signaling in modulation of the immune response after central nervous system trauma

There is growing literature that indicates that members of several classes of SEMAs (including SEMA3s) and their receptors are key modulators of the immune response (reviewed in Mizui et al., 2009; Takamatsu and Kumanogoh, 2012; Kumanogoh and Kikutani, 2013), hence they constitute a family of immunoregulatory molecules that are collectively termed “immune SEMAs”. A variety of mature and differentiating immune cells are susceptible to SEMA3s signaling pathways that drive biological processes as diverse as cell migration, apoptosis and modulation of the immune response at the level of cytokine release. The trauma-induced expression of SEMAs may therefore drastically influence the immune response. This part of the review highlights the role of SEMA3s and does not include the well-documented role of membrane-bound SEMAs in the immune response (reviewed in Chavarría and Cárdenas, 2013; Kumanogoh and Kikutani, 2013).

Traumatic CNS injury initiates a profound immune response in which many molecules and cells are involved (reviewed in Donnelly and Popovich, 2008; Hawthorne and Popovich, 2011). Astrocytes (reviewed in Pekny et al., 2014) and microglia (reviewed in London et al., 2013) are among the first neuroglial cells in the CNS that respond to trauma. The activated astrocytes and microglia release pro-inflammatory cytokines that are toxic for neural tissue. In addition to glial cells, neurons and oligodendrocytes (OLs) synthesize and secrete chemokines and cytokines after trauma (reviewed in Chavarría and Cárdenas, 2013). In the acute phase of injury proinflammatory cytokines such as interleukins (IL-1, IL-6) and tumor necrosis factor (TNFα), are highly expressed, while the “anti-inflammatory cytokines” such as IL-4, TGF-β and reactive oxygen species are expressed in the chronic stages (reviewed in Vidal et al., 2013). The infiltration rate of inflammatory cells is enhanced due to the heavily disrupted BBB/BSCB barrier after traumatic CNS injury (reviewed in Engelhardt and Coisne, 2011).

An elegant flow cytometry and immunohistochemical analysis characterized the cellular inflammatory response over time after SCI in rats (Beck et al., 2010). Neutrophils (e.g., granulocytes and monocytes) are the first inflammatory cells that enter the spinal cord at the day of insult. Monocyte-derived macrophages start to infiltrate the injury site at 3 to 7 days post injury. T-cells enter the injured spinal cord during the second week of injury. In the chronic phase of the immune response (>14 days), microglia/macrophages and lymphocytes are the most abundant inflammatory cell type present in the spinal cord (Beck et al., 2010). A study with human post-mortem tissue showed that a similar chronologic order of cellular events occurs in patients suffering from SCI, and that a lesion-induced immune response could be observed years after trauma (Fleming et al., 2006). In summary, microglia and neutrophils are the main cellular players in the acute phase, while microglia, macrophages and lymphocytes are more dominantly involved in the later sub-chronic and chronic phases of the immune response.

The role for the immune response after CNS trauma is complex and is a topic of intense study. There are experimental studies that addressed the role of the immune response by modulation of cytokines (Lacroix et al., 2002; Ferguson et al., 2008; Genovese et al., 2008; Zhou et al., 2009; Arnold and Hagg, 2011; White et al., 2011; Sato et al., 2012) while other laboratories targeted cellular components to stimulate repair following traumatic CNS injury (Chernykh et al., 2010; Pineau et al., 2010; Wu et al., 2012; Bachstetter et al., 2013; Shechter et al., 2013; Bartus et al., 2014). The part of review below will illustrate that semaphorin signaling could be a target to modulate the immune response after traumatic CNS injury. The injury-induced SEMA3s influence the release of various cytokines and cell types that take part in the immune response.

Macrophages are classified into two distinct populations, which have opposing effects on neuronal tissue. The classical pro-inflammatory type 1 macrophages (M1) secrete cytokines that can be toxic for CNS neurons, while the non-classical anti-inflammatory type 2 macrophages (M2) can promote neuroregeneration across neuronal repulsive substrates (reviewed in David and Kroner, 2011). However, most macrophages in the injured spinal cord are considered to be M1 macrophages (Kigerl et al., 2009). SEMA3A influences the behavior of monocytes and monocyte-derived macrophages. Cell migration assays have demonstrated that SEMA3A inhibits the mobility of human monocytes and a cell type derived from a B-cell lineage *in vitro* (Delaire et al., 2001), an effect that was not observed for SEMA3F. Interestingly, the above study suggested that SEMA3A could signal via yet unidentified receptors to reduce the migration of monocytes since expression of NRP1, the SEMA3A binding receptor, was not detected. However, it should be taken into account that the study was conducted at a time where many receptor components of SEMA3 signaling were not fully identified yet, and the tools to identify them were limited. Furthermore, a more recent *in vitro* study demonstrated that differentiation of human monocytes into M2 macrophages induces the expression of NRPs, PLXNA1, PLXNA2, and PLXNA3 (Ji et al., 2009). Notably, SEMA3A did not affect the migration and phagocytic efficiency of these M-CSF derived M2 macrophages but it did initiate cell death. In contrast, interferon-γ (IFNγ) driven differentiation of monocytes into M1 macrophages down-regulates NRP1 *in vitro* (Ji et al., 2009) and therefore M1 macrophages may not be responsive to SEMA3A.

Thus, SEMA3A could induce apoptosis of monocyte-derived M2 macrophages and inhibit the migration of their progenitor cells *in vitro*. However in a demyelination model *in vivo*, the above pro-apoptotic effects of SEMA3A on the macrophage response were not validated (Syed et al., 2011). Administration of exogenous Sema3A did not affect the phagocytic efficiency and amount of macrophages after a demyelination lesion. Although a distinction in M1 or M2 macrophage subtypes was not made in the latter study. Furthermore one can argue that this demyelination model does not resemble a classical traumatic CNS injury model due to the absence of BBB/BSCB breakdown. It would however be interesting to determine whether Sema3A alters the macrophage heterogeneity at the lesion site by selectively inducing apoptosis of M2 macrophages expressing NRPs.

Other studies showed that lymphocytes, which are suggested to limit functional recovery after CNS trauma (Wu et al., 2012), are also influenced by SEMA3s. Firstly, Sema3A, Sema3E and the receptors NRP1 and PLXND1 mediate the T-cell migration during T-cell maturation in the thymus (Lepelletier et al., 2007; Choi et al., 2008 and reviewed in Mendes-da-Cruz et al., 2012a,b). Although, it is unlikely that trauma-induced expression of SEMA3s in the CNS influences T-cell maturation in the thymus, one could argue that SEMA3s may repel T-cells at the injury site. Secondly, SEMA3A secreted from tumor cells reduces the proliferation of recruited T-cells and antagonizes the synthesis of cytokines such as IL-2, IL-4, IL-10 and IFNγ *in vitro* (Catalano et al., 2006) The inhibitory effect of SEMA3A on T-cells are mediated by the activation of the member of RAS oncogene family Rap1, which in turn, inhibits the phosphorylation of MAP kinase-ERK kinase (MEK) and the mitogen-activated protein kinase (ERK1/2) (Catalano et al., 2006). Thus, SEMA3s inhibit the proliferation of recruited T-cells and decreases cytokine production.

The chronic immune response is initiated when DCs present antigens to T-cells in secondary lymphoid organs. The physical interaction between DCs and T-cells are mediated through homophilic binding of NRP1 receptors. Blockage of NRP1 at both cells interferes with the cell-cell contact and thereby reduces T-cell proliferation (Tordjman et al., 2002). Hence, NRP1 receptors play a major role in the initiation of the primary immune response. Furthermore, SEMA3A signaling via NRP1 inhibits the dendritic-contact mediated T-cell proliferation. SEMA3A impairs the polarization of the actomyosin cytoskeleton and T-cell receptors (TCR) at the DC-T cell contact zone. Moreover, SEMA3A inhibits TCR mediated activation signals such as phosphorylation of the zeta-chain associated protein kinase 70kDa (ZAP-70) and focal adhesion kinase (FAK; Lepelletier et al., 2006). Consequently, SEMA3A negatively regulates T-cell activation and proliferation. In line with this observation, blockage of SEMA3A function results in increased T-cell proliferation (Catalano et al., 2006; Lepelletier et al., 2006). Finally, mice that lack PLXNA4, Sema3A, or contain a NRP1 mutant that is not capable to bind Sema3s, show a hyper proliferative T-cell and immune response after experimental autoimmune encephalomyelitis (Yamamoto et al., 2008). Thus, trauma-induced expression of SEMA3s may therefore inhibit the T-cell response at the site of injury suggesting that blockage of Sema3A signaling could potentially enhance it.

SEMA3A influences DCs, which in turn, are important for initiation of the immune response. SEMA3A modulates DCs through NRP1 and PLXN signaling. A knock-out study showed that PLXNA1 at DCs is required for normal T-cell activation and their synthesis of cytokines IFNγ, IL-2 and IL-4 (Takegahara et al., 2006). Furthermore, Sema3A signaling via PLXNA1 allows DCs to enter the lymphatic system *in vivo*. Sema3A acts on the rear side of DCs, enhances their migration speed and allows DCs to pass EC-junctions by actomyosin contraction *in vitro* (Takamatsu et al., 2010). Signaling via PLXNB2 and PLXND1 receptors did not affect DC migration, but down-regulated the synthesis of pro-inflammatory cytokine IL-12/IL-23p40 (Holl et al., 2012). Thus, SEMA3s stimulates the migration of DCs into the lymphatic system and influences their synthesis of cytokines.

Activated microglia are also sensitive to Sema3A. IFNγ mediated activation of microglia *in vitro* induces the expression of the Sema3A receptors NRP1 and PLXNA1, while their exposure to exogenous Sema3A induces apoptosis in a NRP1 dependent manner (Majed et al., 2006). The Sema3A mediated apoptosis was validated in a model of toxin-induced focal brain injury *in vivo*, where stressed neurons secreted Sema3A and microglia upregulated NRP1 after injury. The apoptotic microglia co-localized with Sema3A *in vivo* (Majed et al., 2006), indicating that Sema3A released from stressed neurons could induce death of injury-activated microglia. There is controversy whether microglia are beneficial or deleterious for recovery. Microglia can secrete reactive oxygen species and pro-inflammatory cytokines that can harm neurons, but may on the other hand have neuroprotective roles, i.e., by phagocytic clearance of toxic molecules and myelin debris (reviewed in London et al., 2013).

Platelet cells are involved in the acute immune response by neutralizing bacteria and they stimulate the activation of neutrophils and DCs. Next to cell-cell interactions, platelet cells secrete granules and cytokines that contribute to these processes. For example, platelet cells contain high levels of the pro-inflammatory IL-1β, anti-inflammatory cytokine TGF-β and stimulate the DC-mediated synthesis of anti-inflammatory IL-10 (reviewed in Semple et al., 2011). Platelet cells are sensitive to SEMA3s through their expression of NRP1 and PLXNA receptors (Kashiwagi et al., 2005). Sema3A inhibits platelet functions including, granular secretion, adhesion to FN and platelet spreading. This is achieved by inactivation of αIIβ3 integrin and the Rho GTPase (RacI)-mediated cytoskeleton rearrangements (Kashiwagi et al., 2005). To date, it is unknown whether SEMA3A affect platelet cells *in vivo* following CNS trauma.

There are a number of competitors of SEMA3A signaling in the immune system. NRPs are co-receptors for multiple ligands such as VEGFs and TGF-β that compete for binding sites. VEGFs enhance the permeability of blood vessels (reviewed in Olsson et al., 2006) and therefore contribute to the infiltration of inflammatory cells to the lesion site. Furthermore, VEGFs are direct modulators of the immune response. Contrary to SEMA3s, VEGFs act as pro-inflammatory cytokines by recruiting monocytes and macrophages and activate them by inducing the secretion of granulocyte-macrophage colony stimulating factor (GM-CSF). At chronic stages, VEGFs can suppress the immune response by inhibiting the maturation of DCs and impairing the function of T-cells (reviewed in Vitale et al., 2010). After traumatic CNS injury, VEGFA is highly expressed for a relative short period (Sköld et al., 2000; Vaquero and Zurita, 2004; Herrera et al., 2009), and may therefore promote the immune response in the acute phase of injury.

TGF-β is another key regulator of the immune response and modulates the cell proliferation, differentiation, migration and survival properties of immune cells. In the chronic stages of injury, the role of this cytokine is mainly to suppress the immune response. For instance, TGF-β influences macrophages by inhibiting their activation, phagocytic efficiency and antigen presentation capacity (reviewed in Li et al., 2006). Furthermore, the interaction with NRP1 suppresses T-cells by inhibiting their proliferation and production of pro-inflammatory cytokines like IL-2 *in vitro* (Glinka and Prud’homme, 2008). TGF-β is highly expressed after traumatic SCI and remains elevated up to a year in humans (Buss et al., 2008). Therefore, the functional competition among SEMA3s and TGF-β for binding to NRP1 appears to be of particular interest.

Several studies have suggested that TGF-β can signal through its canonical (Smad2/3) signaling, which is NRP1-independent, exerting anti-proliferative and immunosuppressive effects that induce fibrosis and non-canonical signaling pathway which is NRP1-dependent and can antagonize canonical signaling (reviewed in Prud’homme and Glinka, 2012). It is therefore reasonable to assume that blocking SEMA3 activity might be beneficial for tissue sparing by allowing NRP epitopes to interact with this growth factor and therefore favoring the TGF-β non-canonical signaling pathway. Thus, TGF-β signaling via NRPs may contribute to control the immune response in chronic stages of injury. Collectively, signaling of VEGFs and TGF-β via NRP1 modulates the immune response after traumatic injury. VEGFA may boost the immune response which could harm CNS neurons in the acute phase of injury, while TGF-β suppresses the immune response in chronic stages of injury.

Taken together these data indicate that SEMA3s influence the function of many inflammatory cells including monocytes, B-lymphocytes, M2 macrophages, T-lymphocytes, activated microglia, DCs and platelet cells. SEMA3 signaling results in modification of the cytokine profile and cellular components of the immune response. During the acute inflammatory response SEMA3s can inhibit the recruitment of monocytes and B-cells and neutralize the functions of platelet cells. Furthermore they may stimulate DCs and steer them away from the site of injury. On the other hand at sub-chronic and chronic stages of injury SEMA3s may restrict an extensive immune response. As mentioned earlier in this review, Sema3A did induce apoptosis of neuroprotective M2 macrophages and activated microglia. Furthermore, Sema3A was able to suppress *in vitro* the proliferation of recruited T-lymphocytes and inhibit the synthesis of their cytokines. Whether these functions occur *in vivo* as well is yet to be elucidated. In this context, it is plausible that SEMA3s control the inflammation response both at acute and chronic stages. The question remains though, whether the immune response is beneficial or detrimental for recovery after traumatic CNS injury.

A possible consequence of SEMA3s expression after CNS injury is the masking of NRP1 epitopes that could interact with other ligands like TGF-β. TGF-β can suppress the immune response at chronic stages by controlling virtually all-immune cells. From this point of view, SEMA3s may interfere with TGF-β signaling on the inflammatory cells in scar tissue. Blockage of class III “immune SEMAs” after CNS injury may therefore result in unwanted elevated level of the immune response due to the loss of SEMA3s function. However two potential mechanisms might counteract these undesired effects: (1) As mentioned earlier in this review blockage of SEMA3s signaling might favor revascularization and thus the innate ability of the organism for effective clearance and recycling of the excessive inflammatory cells through the circulatory pathway; and (2) NRP1 receptors of inflammatory cells may become available for TGF-β signaling, which suppress the immune response perhaps more sufficiently than SEMA3s. Therefore, the role of SEMA3s in modulating the immune response after traumatic CNS injury needs to be investigated in more detail.

## The role of semaphorin signaling in re-myelination

Trauma of the CNS induces mechanical forces that cause loss of OLs and demyelination of axons at and surrounding the injury site. The loss of myelin sheaths decreases the conduction velocity of action potentials and could result in axonal degeneration. The reestablishment of myelin sheets around demyelinated axons and around newly sparsely formed regenerating axons, a process called remyelination, is therefore important to regain neuronal function after brain and spinal cord injuries (reviewed in Franklin and Ffrench-Constant, 2008). Traumatic CNS injury leads to deposits of myelin debris around the scar and in the denervated nerve tracts, which leads to additional impairment of functional recovery. Firstly, myelin-derived axon repulsive molecules such as Nogo-A, MAG and OMgp, as well as a number of secreted and membrane bound SEMAs, restrict regrowth of the injured axons (reviewed in Schwab, 2004; Xie and Zheng, 2008; Lee and Zheng, 2012). Secondly, myelin debris limits remyelination by inhibiting the differentiation of oligodendrocyte precursor cells (OPCs) into new functional OLs (Kotter et al., 2006). Thus, traumatic CNS injury leads to pathology of the white matter and restricts regenerative processes such as axon regrowth and differentiation of OPCs.

Re-myelination does occur in healthy and injury conditions of the adult CNS. Adult born OLs synthesize new myelin sheaths but contain shorter and thinner internodes compared to post-natal born OLs (Hughes et al., 2013; Young et al., 2013). The typical adult born OL derived myelin sheaths are observed on demyelinated axons that survive SCI at chronic stages post-injury (Smith and Jeffery, 2006; Lasiene et al., 2008). After traumatic CNS injury, two endogenous mechanisms are initiated to remyelinate surviving axons. Firstly, SCI causes proliferation of OPCs and adult-born OLs initiate remyelination of the naked axons (McTigue et al., 2001). However, the oligogenesis is limited to the reactive lesion border after injury (Tripathi and McTigue, 2007). Secondly, Schwann cells from the peripheral nerves and neuronal crest precursor cells undergo plasticity changes and migrate to the spinal cord lesion (Nagoshi et al., 2011). These Schwann cells provide neurotrophic support and participate in the re-myelination of surviving axons. Taken together, remyelination occurs at demyelinated CNS axons that survive traumatic injury, it is however limited to the peri-lesion neuropil.

Remyelination in the adult CNS requires the activation and recruitment of OPCs and their differentiation into mature OLs. Many extrinsic factors, including the immune response, modulate the function of OPC in the injured CNS. SEMA3s are one of the molecules known to influence the oligodendrocyte lineage and re-myelination (reviewed in Kotter et al., 2011). Semaphorins like SEMA3A and SEMA3F are upregulated in demyelinated lesions of MS patients and experimental animal models (Williams et al., 2007). Oligodendrocytes and their precursor cells express NRPs (NRP1 and NRP2) and a wide range of PLXNs, indicating that these cell types are sensitive to SEMA3s (Cohen et al., 2003; Okada et al., 2007; Piaton et al., 2011; Syed et al., 2011; Xiang et al., 2012; Boyd et al., 2013). Furthermore, demyelination induces an upregulation of these semaphorin receptors in OPCs *in vivo* (Piaton et al., 2011). Therefore it is well documented that the oligodendrocyte lineage has the ability to respond to injury-induced SEMA3s in the adult CNS.

One of the potential mechanisms by which SEMA3s act is that they modulate the guidance of the oligodendrocyte lineage. Stripe and outgrowth assays have demonstrated that cultured OPCs are not capable to migrate across high concentrations of Sema3s, including Sema3A, Sema3B, Sema3C and Sema3F (Cohen et al., 2003). The same study showed that Sema3B and Sema3C also repel and collapse processes of cultured OPCs. Another study demonstrated that Sema3A is repulsive for OPC migration, while Sema3F enhances the proliferation of embryonic OPCs and has an attractive effect *in vitro* (Spassky et al., 2002). The effect of Sema3A and SEMA3F on OPC migration was confirmed in a more recent study, in which OPCs were isolated from the adult brain and spinal cord (Piaton et al., 2011). The migration of OPCs by Sema3F is mediated by NRP2 and PLXNA3 signaling *in vitro* (Xiang et al., 2012). Collectively these data indicate that most SEMA3s block OPC migration, in contrast to SEMA3F that accelerates their migration *in vitro*.

In line with the *in vitro* studies, the important role of SEMA3s in OPCs migration was validated *in vivo*. SEMA3A and SEMA3F have been increasingly implicated to influence the recruitment of OPCs and remyelination. In post-mortem tissue, high expression levels of SEMA3A in MS lesions correlated with low numbers of OPCs (Boyd et al., 2013). Furthermore, the migration of OPCs in the CNS has been modified in animal models by changing the relative expression of Sema3A and Sema3F. More precisely, viral delivery of Sema3A prior to a demyelination lesion had a repulsive effect on OPCs, in contrast to Sema3F that attracted OPCs (Piaton et al., 2011). Similarly, injection of recombinant Sema3A or Sema3F after induction of a white matter lesion lead to comparable phenotypes (Boyd et al., 2013). Furthermore, blockage of Sema3A signaling enhances the recruitment of OPCs, as validated in two transgenic mice models: (1) NRP1 mutated in the Sema3 domain (Piaton et al., 2011); and (2) knockdown of endogenous Sema3A (Boyd et al., 2013). Boyd et al. showed that reduced expression of Sema3A promoted re-myelination *in vivo*. Thus, Sema3A signaling inhibits OPC recruitment in demyelinated lesions and can impair remyelination. In contrast, Sema3F mediated OPC recruitment promoted remyelination in the lesion (Piaton et al., 2011; Boyd et al., 2013). Taken together, Sema3A is a negative regulator of OPC recruitment while Sema3F facilitates OPC recruitment in the demyelinated areas *in vivo*. This highlights that stimulation of OPC recruitment by manipulating the expression of these two SEMA3s could promote functional remyelination.

Another potential mechanism for SEMA3s to block remyelination is by inhibition of OPC differentiation. Application of Sema3A to cultured primary OPCs inhibited their differentiation into OLs. Conveniently, the effects of Sema3A on OPC differentiation were dose-depend and reversible (Syed et al., 2011). This clearly demonstrates that SEMA3A acts as inhibitor of OPC differentiation in *vitro*. SEMA3A could therefore potentially block differentiation of OPC after demyelination *in vivo*. Correspondingly, administration of Sema3A ten days after a demyelination lesion in the adult CNS influenced OPCs and remyelination. The observations made from Syed et al. indicate that delayed delivery of Sema3A does not alter the migration but inhibits OPC differentiation in demyelinated lesions. To add more to this evidence, a strong impairment of remyelination was observed in Sema3A infused lesions (Syed et al., 2011). Based on these recent findings, it is clear that SEMA3A inhibits CNS remyelination and that one of the mechanisms is the arrest of the differentiation of OPC at a pre-myelinating state *in vivo*.

SEMA3A also influences the migration of the alternative type of myelinating cells in the CNS, the Schwann cells. The study from Kaneko et al. (2006) documented that Schwann cells are responsive to SEMA3A by the expression of NRP1, PLXNA1 and PLXNA4. Furthermore, stripe assays demonstrated that Sema3A has a repulsive effect on the migration of Schwann cells. Consistent with the *in vitro* findings, the *in vivo* observations revealed that this repulsive effect of Sema3A on Schwann cells is suppressed by Xanthofulvin treatment (Kaneko et al., 2006). It is known that after SCI in adult rat, Schwann cells are capable to migrate from the periphery to the injury site and start remyelination (Nagoshi et al., 2011). Xanthofulvin treatment in the injured spinal cord indeed promoted extensive Schwann cell migration and remyelination in the lesion site (Kaneko et al., 2006). This suggests yet another mechanism by which SEMA3A could inhibit CNS remyelination and that is via inhibition of Schwann cell migration *in vivo*.

SEMA3s are not the only ligands of NRPs that have a role in remyelination. The extracellular domain of NRP1 can form a receptor complex with EGFRs on the cell surface of tumor cells. NRP1 regulates clustering of EGFRs and elevates the activity of their signaling pathway (Rizzolio et al., 2012). It seems plausible that NRP1 forms complexes with EGFRs at the oligodendrocyte lineage as well. A study that used a gain and loss of EGFR function approach showed that EGFR signaling plays a significant role in remyelination in adult mice (Aguirre et al., 2007) by promoting: (1) proliferation of OPCs, (2) migration into the white matter lesion, (3) adult oligogenesis and importantly (4) axon remyelination. Appropriately, these pro-remyelination effects were decreased in transgenic mice with hypoactive EGFR signaling, as demonstrated in the same study. Hence, enhancing EGFR signaling could be a strategy to target the regenerative processes of remyelination after CNS trauma. As mentioned above, SEMA3A signaling via NRP1 seems to have opposing effects on remyelination compared to EGFR. However, it is unknown whether there is functional competition between both ligands for NRP1 binding on OLs and if this may influence remyelination.

PDGF is one of the critical growth factors that regulate OPC proliferation, survival and migration of the OL lineage. PDGF is therefore suggested to be important for the distribution of OPCs during development and adulthood (reviewed in Mitew et al., 2014). PDGF signaling could also have a clinical relevant role since the PDGF alpha receptors (PDGFαR) are expressed at OPCs in healthy white matter and active MS lesions of humans (Wilson et al., 2006). Thus, PDGF signaling on OPCs is associated with remyelinating areas in the adult CNS of humans. Interestingly enough, NRP1 has been shown to mediate PDGF signaling in various cell types including: hepatic satellite cells (Cao et al., 2010), mesenchymal stem cells (Ball et al., 2010) and vascular smooth muscle cells (Banerjee et al., 2006; Pellet-Many et al., 2011). As described earlier, NRPs are expressed in OPCs and play a significant role in re-myelination. The above studies do not rule out the possibility that NRP1 may be acting via yet another non-classical binding partner. Hence, NRP1 dependent signaling of SEMA3s may additionally counteract PDGF induced OPC survival, proliferation and migration.

Taken together the above experimental data suggest that SEMA3s modify the function of the oligodendrocyte lineage. Multiple SEMA3s inhibit OL migration *in vitro*. Furthermore, Sema3A has been shown to inhibit remyelination by blocking the recruitment of OPCs and their differentiation into myelinating OLs and could also inhibit the recruitment of Schwann cells. In contrast to the inhibitory functions of most SEMA3s, SEMA3F stands out as the only secreted semaphorin that exerts chemo-attractive properties for OPCs with the potential to promote remyelination. Future studies should focus on targeting specific SEMA3s or their receptors as a potential approach to improve re-myelination. NRP1 could be targeted to enhance remyelination by inhibiting the function of Sema3A. Blockage of Sema3A signaling may be beneficial for remyelination by stimulating OPC recruitment and differentiation at the neural scar. Furthermore, interference with SEMA3A may facilitate EGF and PDGF signaling via NRP1 and thereby promote remyelination. A selective inhibitor of SEMA3A-NRP1 signaling would not affect the pro-myelinating effects of SEMA3F since it signals mainly via NRP2. Hence, injury-induced SEMA3F may still promote OPC migration in the lesion site and promote remyelination. The evidence accumulated from multiple studies in demyelination lesions, proposes that alternative strategies to promote remyelination could focus on enhancing SEMA3F mediated OPC migration, which in combination with boosting EGF and PDGF signaling might result in sufficient remyelination. Finally, based on research in the field of MS, it has been suggested that the acute immune response could promote re-myelination but this function may be restricted in later stages (reviewed in Kotter et al., 2011). The high expression of SEMA3s after 2 weeks of traumatic injury correlates strongly with restricted remyelination.

## Conclusions

**Figure 1:**
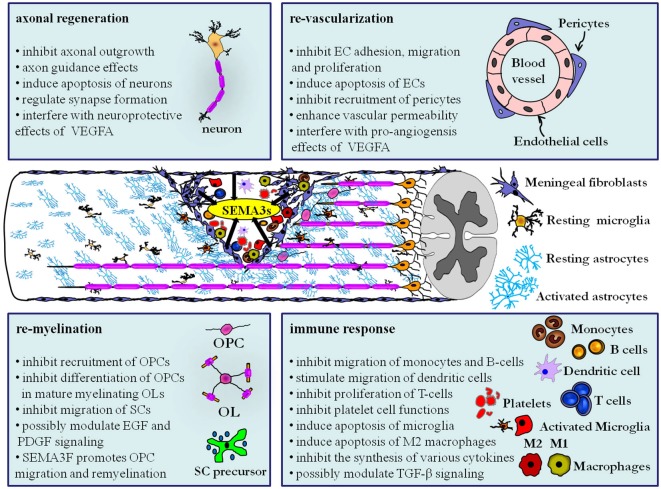
**Class III semaphorins exert regulatory functions in multiple processes after CNS trauma by modulating various neuronal and non-neuronal cell types**. Deposition of SEMA3s (black arrows) by invading fibroblasts from the ruptured meningeal layer and axotomized neurons has a large consequence for cellular remodeling and wound healing. The secreted SEMA3s have a wide range of biological effects on the resident glial cells and neurons, and additionally affect the blood derived cells that infiltrate the lesion core as a result of blood vessel rupture. As discussed in this review, the role of SEMA3s goes beyond inhibiting axonal regeneration and could be a significant target for future studies to stimulate repair following CNS trauma. Abbreviations: B-cell, B-lymphocyte; EC, endothelial cell; EGF, epidermal growth factor; OL, Oligodendrocytes; OPC, oligodendrocyte precursor cells; PDGF, platelet-derived growth factor; SC, Schwann cells; T-cell, T-lymphocyte; TGF-β, transforming growth factor—beta.

This review outlined the functions of SEMA3s and their receptors in key biological processes that play a prominent role following traumatic CNS injury, including (1) axonal regeneration; (2) re-vascularization; (3) immune response; and (4) re-myelination (Figure 1). SEMA3s and their receptors can affect the aforementioned processes in both the acute and sub-chronic/chronic stages of traumatic CNS injury. In the acute phase of CNS injury local expression of SEMA3s may negatively regulate axonal regeneration by interfering with the neuroprotective functions of VEGFA, re-vascularization by directly regulating EC adhesion, migration, proliferation and survival or by suppressing the pro-angiogenesis effects of VEGFA, immune response by inhibiting the mobility of monocytes, B-lymphocytes and possibly by silencing the pro-inflammatory effects of platelet cells, and re-myelination by arresting close-by OPCs in a premature stage before chronic stages of injury is initiated.

In the sub-chronic and chronic phase of CNS injury SEMA3s may inhibit axonal regeneration by inducing apoptosis of neurons and by preventing axons to penetrate scar tissue. They may also restrict re-vascularization by SEMA3B inhibition of the recruitment of pericytes that normally stabilize new blood vessels and by SEMA3A mediated elevation of the vascular permeability of blood vessels even when the expression of VEGFA declines. This increased vascular permeability may have a direct impact on the chronic immune response where SEMA3s are shown to inhibit the proliferation of recruited T-lymphocytes and antagonize the synthesis of cytokines such as IL-2, IL-4, IL-10 and IFNγ. Furthermore SEMA3A induces apoptosis of microglia that express NRP1 and thereby influence the survival and axonal regeneration capacity of injured CNS neurons. Additionally SEMA3A induces cell death of M2 macrophages and could thereby induce a “pro-inflammatory” M1 macrophage environment in the scar. An intriguing but yet not fully established hypothesis is that SEMA3s might modulate TGF-β non-canonical signaling via NRPs at inflammatory cells near the scar tissue. Finally, re-myelination is also affected by SEMA3s at the chronic stages of injury. In particular SEMA3A inhibits the recruitment and differentiation of OPCs and inhibits the mobility of migrating Schwann cells. In contrast, SEMA3F promotes OPC migration and remyelination. Lastly, SEMA3A signaling via NRP1 might antagonize furin-processed SEMA3F, EGF and PDGF signaling pathways that all posses the ability to stimulate remyelination.

## Perspectives

Since the discovery of Sema3A in 1993, SEMA3s signaling and their receptors were increasingly recognized to be involved in key processes influencing axonal outgrowth and guidance. However, substantial evidence indicates that SEMA3s also participate in cell-to-cell communication systems that underlie angiogenesis, inflammation and re-myelination. It will be necessary to combine the knowledge obtained from the research fields of vascular biology, cancer biology, immunology and glial biology in order to better understand the role of SEMA3s signaling in CNS trauma. A combinatorial approach involving these fields of research may be necessary in order to move closer towards a therapy for traumatic CNS injury. SEMA3s are an important target in strategies that aim to repair traumatic spinal cord and brain injuries, however the sometimes overlooked pleiotropic nature of their NRP receptors needs to be addressed/understood in a more thorough fashion in future studies.

## Conflict of interest statement

The authors declare that the research was conducted in the absence of any commercial or financial relationships that could be construed as a potential conflict of interest.
